# Identification of Nucleases and Phosphatases by Direct Biochemical Screen of the *Saccharomyces cerevisiae* Proteome

**DOI:** 10.1371/journal.pone.0006993

**Published:** 2009-09-15

**Authors:** Chu Kwen Ho, Alicia F. Lam, Lorraine S. Symington

**Affiliations:** Department of Microbiology, Columbia University College of Physicians & Surgeons, New York, New York, United States of America; University of Minnesota, United States of America

## Abstract

The availability of yeast strain collections expressing individually tagged proteins to facilitate one-step purification provides a powerful approach to identify proteins with particular biochemical activities. To identify novel exo- and endo-nucleases that might function in DNA repair, we undertook a proteomic screen making use of the movable ORF (MORF) library of yeast expression plasmids. This library consists of 5,854 yeast strains each expressing a unique yeast ORF fused to a tripartite tag consisting of His_6_, an HA epitope, a protease 3C cleavage site, and the IgG-binding domain (ZZ) from protein A, under the control of the *GAL1* promoter for inducible expression. Pools of proteins were partially purified on IgG sepharose and tested for nuclease activity using three different radiolabeled DNA substrates. Several known nucleases and phosphatases were identified, as well as two new members of the histidine phosphatase superfamily, which includes phosphoglycerate mutases and phosphatases. Subsequent characterization revealed YDR051c/Det1 to be an acid phosphatase with broad substrate specificity, whereas YOR283w has a broad pH range and hydrolyzes hydrophilic phosphorylated substrates. Although no new nuclease activities were identified from this screen, we did find phosphatase activity associated with a protein of unknown function, YOR283w, and with the recently characterized protein Det1. This knowledge should guide further genetic and biochemical characterization of these proteins.

## Introduction

**Figure 1:**
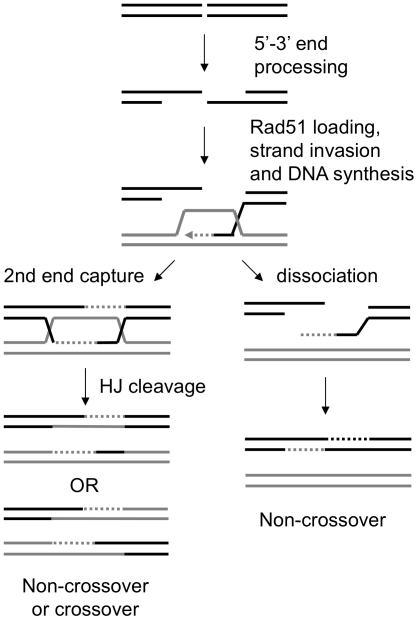
Model of DSB repair by homologous recombination. After formation of a DSB, helicases and nucleases promote 5′–3′ resection of DNA ends to generate long 3′ ssDNA tails. The ssDNA tails are the substrate for binding by Rad51 to promote strand invasion and pairing with a homologous duplex. The 3′ end of the invading strand initiates DNA synthesis and is displaced by helicases to pair with the other side of the break to generate non-crossover products. Alternatively, after DNA synthesis the second end is captured, forming a double Holliday junction intermediate. These junctions are resolved by HJ resolvase(s) to generate crossover or non-crossover products, depending on orientation of cleavage of the junctions by resolvase. Alternatively, these junctions are dissolved by BLM-TopoIIIα-RMI1 helicase-topoisomerase complex to generate non-crossover products.

Nucleases play essential roles in nucleic acid metabolism. The proofreading exonucleases associated with the replicative DNA polymerases, and structure-specific endonucleases that process Okazaki fragments, are essential for the fidelity and completion of DNA synthesis, respectively [1]. Endo- and exonucleases are critical for incision of the DNA backbone adjacent to damaged bases and excision of nucleotides during repair [2]. Nucleases function at several steps in the homologous recombination (HR) pathway for double-strand break repair. Exo- and endonucleases function in the initiation of HR by degrading DNA ends to produce 3′ single-stranded DNA tails that are bound by the Rad51 recombinase [3]. Following strand invasion and DNA synthesis, recombination intermediates containing Holliday junctions must be resolved to allow segregation of the repaired DNA duplexes (Fig. 1). Resolution of HJs is predicted to require the activity of structure-specific endonucleases.

Yeast has proved a valuable system for the analysis of HR in eukaryotes. Much of our understanding of the mechanisms of HR is based on physically monitoring double-strand break-induced recombination in *Saccharomyces cerevisiae* and analysis of mutants defective for different steps in the pathway. However, until recently the identity of the nucleases functioning in the earliest step of recombination, 5′–3′ resection of DSBs, was unknown because of redundancy for this step of the pathway [4], [5], [6]. Similarly, no single mutant with the predicted resolution-defective phenotype has been identified in budding yeast, suggesting there is also redundancy for processing HJ-containing recombination intermediates. The Mus81-Mms4 (Mus81-Eme1 in fission yeast) heterodimeric nuclease resolves strand invasion intermediates by two sequential cleavages of branched intermediates to form crossover products. This mode of resolution is essential for meiotic recombination in *S. pombe*, but plays a less important role in *S. cerevisiae* [7], [8], [9], [10]. The human Bloom's syndrome helicase complex (BLM-TopoIIIα−ΡΜΙ1) has been shown to resolve dHJ intermediates *in vitro* by a process called dissolution [11], [12], [13]. The helicase activity of BLM branch migrates the constrained HJs and the topoisomerase activity of TopoIIIα is thought to remove the supercoils between the two HJs eventually leading to the resolution in a non-crossover configuration (Fig. 1). Biochemical approaches to identify HJ resolvases from fractionated extracts of yeast identified the mitochondrial protein Cce1 (cruciform cleaving endonuclease); this has no obvious function in nuclear HR [14], [15]. An endonuclease activity with specificity for intact HJs has been purified from mammalian cell extracts and recently identified as a member of the Rad2/XPG family of nucleases called GEN1 (Yen1 in budding yeast) [16], [17], [18]; however, there are currently no *in vivo* data to support a role for Yen1/GEN1 in HJ resolution.

Because genetic approaches suggest there are redundant HJ resolving activities, and possibly other exo- or endonucleases involved in HR, we undertook a proteomic screen making use of the movable ORF (MORF) library of yeast expression plasmids [19]. This library consists of 5,854 yeast strains each expressing a unique yeast ORF fused to a tripartite tag consisting of His_6_, an HA epitope, a protease 3C cleavage site, and the IgG-binding domain (ZZ) from protein A, under the control of the *GAL1* promoter for inducible expression. The His_6_ and ZZ domains provide a means for affinity purification of the over-expressed proteins. The MORF library represents 93.2% of the verified *S. cerevisiae* ORFs and most of these have either been sequenced completely or are expected to lack mutations based on the error rates of the polymerases used to amplify the yeast ORFs. This collection of strains has been successfully used to detect glycosylated proteins and the activity of proteins known to catalyze tRNA modification reactions [19]. One possible disadvantage of the over-expression screening approach is that proteins that are part of complexes may not be detected if only one subunit is over-expressed. A collection of yeast strains in which the endogenous locus of individual ORFs has been tagged at the C-terminus with a tandem affinity purification (TAP) tag is also commercially available and has been used to identify components of protein complexes [20]. However, because the proteins in the TAP-tag collection are expressed from the endogenous promoter and are present in single copy, pooling strategies are unlikely to be successful in identification of poorly expressed proteins. Thus, in order to screen for nuclease activities we chose to use the MORF library to accomplish a comprehensive screen of the yeast proteome more rapidly.

## Results

### Identification of Known Nucleases, Helicase and Phosphatases

**Figure 2:**
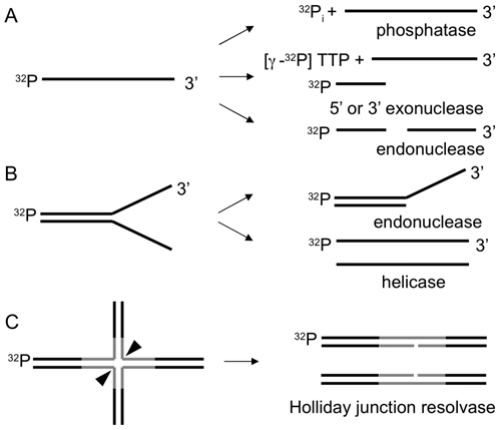
Substrates to assay for nuclease, phosphatase and helicase activities. A. Phosphatases can catalyze the removal of ^32^P_i_ from ssDNA; 5′ to 3′ single-strand specific exonucleases can degrade DNA to release ^32^P-labeled nucleotides, 3′ to 5′ single-strand specific exonucleases degrade the 3′ end resulting in a shortened labeled substrate, or the substrate can be cleaved by endonucleases to generate ^32^P-labeled DNA fragments. B. Structure-specific endonucleases can cleave the single-stranded DNA tail adjacent to duplex region of the Y DNA substrate. In the presence of ATP, helicases can unwind Y substrate to the two constituent ssDNA oligonucleotides. C. Holliday junctions can be cleaved by a resolvase to generate nicked duplex products by introducing paired incisions across the junction.

The *S. cerevisiae* MORF (movable ORF) fusion protein library [19] was screened for proteins, which could degrade and/or cleave ^32^P-labeled ssDNA, Y-shaped and HJ-containing substrates (Figure 2). To conveniently assay 5,854 proteins, the library strains were grown in pools, with each pool containing 24 strains expressing individual fusion proteins. After induction, cells were lysed and proteins were purified by IgG sepharose bead pull down, followed by cleavage with protease 3C. The pools of proteins were then assayed with the 3 different ^32^P-labeled substrates (ssDNA, Y and HJ-X26) for nuclease activities. After activity was observed in one pool, the active pool was deconvoluted to identify the strain and ORF responsible for the activity. For pools that contained a known nuclease, a separate pool was generated of 23 strains without the nuclease to determine whether that nuclease was responsible for the activity within the 24-strain pool. The identity of each ORF was confirmed by the size as determined by gel electrophoresis and Western blotting using HA antibody, and by sequencing of the plasmid DNA recovered from the yeast strain. The latter step was necessary because the plate position for some strains does not correspond to the plate position on the spreadsheet provided by the company (Open Biosystems).

**Figure 3:**
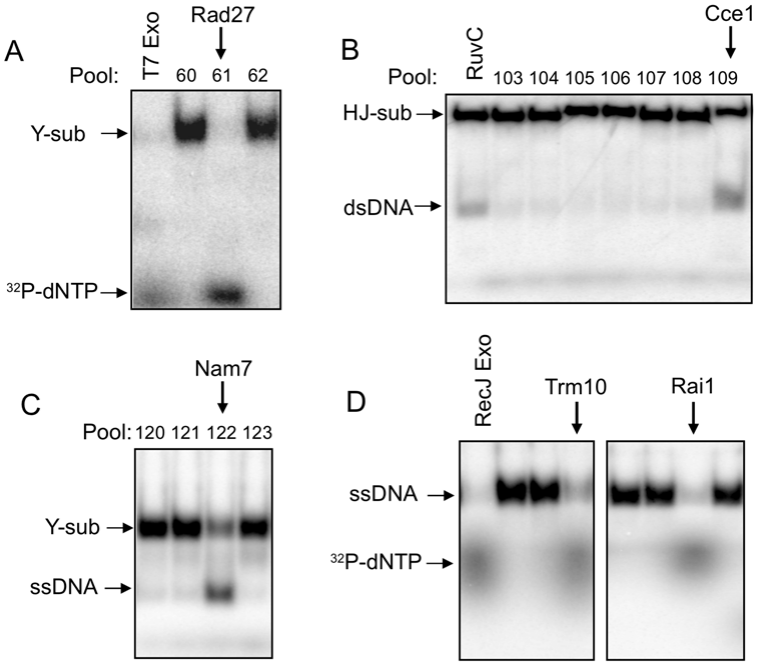
Identification of nucleases and a helicase from the MORF library. Reaction mixtures contained an aliquot of the indicated protein pool or purified from an individual library strain incubated with one of the three radiolabeled DNA substrates (ssDNA, Y or HJ). A. The Y substrate was incubated with T7 exonuclease (control) or three different protein pools (60–62). The activity in pool 61 is due to Rad27. B. The HJ substrate was incubated with *E. coli* RuvC (control) or seven different protein pools (103–109). Pool 109 contains Cce1. C. The Y substrate was incubated with four different protein pools (120–123). The activity in pool 122 is due to Nam7. D. The ssDNA was incubated with *E. coli* RecJ (control), Trm10, Rai1, or other proteins purified from individual library strains (not indicated). Reaction products were resolved by 10% (for Y and HJ) or 15% (for ssDNA) native polyacrylamide gels and analyzed using a phosphorimager. Positions of reaction substrates and products after gel electrophoresis are indicated.

In this screen, the most robust nuclease activity was identified in pool 61, which exhibited nuclease activity on all three radiolabeled DNA substrates (Fig. 3A and data not shown). After deconvolution of the pool and sequencing the plasmid in the active strain, the nuclease was identified as Rad27 (FEN-1). The plate position for Rad27 is inverted compared with the spreadsheet location; another group independently found this error (P. Burgers, personal communication). Several other known nucleases, including Apn1, Pso2, Rat1 and Rex2 were also identified in this screen (data not shown). Like Rad27, the plate position for Pso2 does not match the spreadsheet location. We did not detect activity for other members of the Rad27/FEN-1 nuclease family, Exo1, Din7 and Rad2 in the pools screened, and only a very weak activity was detected for the individual strain expressing Exo1 that was grown and processed separately (data not shown). The mitochondrial HJ resolvase, Cce1, was identified using the ^32^P-labeled HJ substrate (Fig. 3B). We did not detect the recently identified HJ resolvase, Yen1 [18], because this clone is absent from the MORF library. We failed to detect structure-specific nuclease activity for Mus81, Mms4, Rad1 or Rad10-containing pooled extracts. Because these nucleases are functional as heterodimers, and only one subunit is over-expressed, it is likely the amounts of heterodimer are too low to be detected, especially as Y and intact HJs are not the preferred substrates for either Mus81-Mms4 or Rad1-Rad10 [10], [21], [22]. The presence of ATP in the reaction mixtures enabled us to detect a known RNA helicase, Nam7, which unwound ^32^P-labeled Y substrate to the two constituent ssDNA oligonucleotides (Fig 3C). This was the only helicase activity that was detected in the screen.

**Figure 4:**
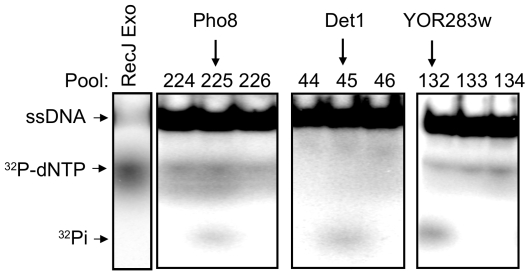
Identification of phosphatases. Reaction mixtures contained ssDNA substrate and an aliquot of the indicated protein pool (224–226, 44–46 and 132–134) or *E. coli* RecJ (control). Protein pool 225, 45 and 132 contained Pho8, Det1 and YOR283w library proteins, respectively. Reaction products were resolved by 15% native polyacrylamide gels and analyzed using a phosphorimager. Positions of ssDNA substrate, released ^32^P-dNTP and ^32^P_i_ after gel electrophoresis are indicated.

In addition to nucleases, we detected phosphatase activity by release of labeled inorganic phosphate from the DNA substrates. The increased electrophoretic mobility distinguished Pi from dNTP released by the RecJ exonuclease. The protein pools containing Pho8 (Fig. 4) and Ptc5 (data not shown), which are reported to exhibit protein phosphatase activity, were identified in the screen [23], [24]. Thus, the high sensitivity of the nuclease assay enabled us to detect protein phosphatases despite the use of DNA substrates in the reactions.

### tRNA Methyltransferase Trm10 is Associated With a Nuclease Activity

Assay and deconvolution of active pool 244 confirmed that ORF YOL093W is associated with a ssDNA exonuclease activity (Fig 3D). YOL093W encodes a protein called Trm10, which was identified in a library screen to be a tRNA methyltransferase [25]. As shown in Figure 3D, Trm10, purified from an individual library strain, could degrade the ^32^P-labeled ssDNA substrate to radiolabeled nucleotides. No processing of the radiolabeled Y or HJ substrates by Trm10 was observed (data not shown). It seems most likely that the nuclease activity is due to an associated protein that co-purifies with Trm10 because Trm10 contains no domains previously associated with nuclease activity. Identification of co-purifying nucleases is possible because Rai1, which interacts with the exoribonuclease Rat1 [26], was found in the screen (Fig. 3D).

### Identification of Two New Phosphatases

**Figure 5:**
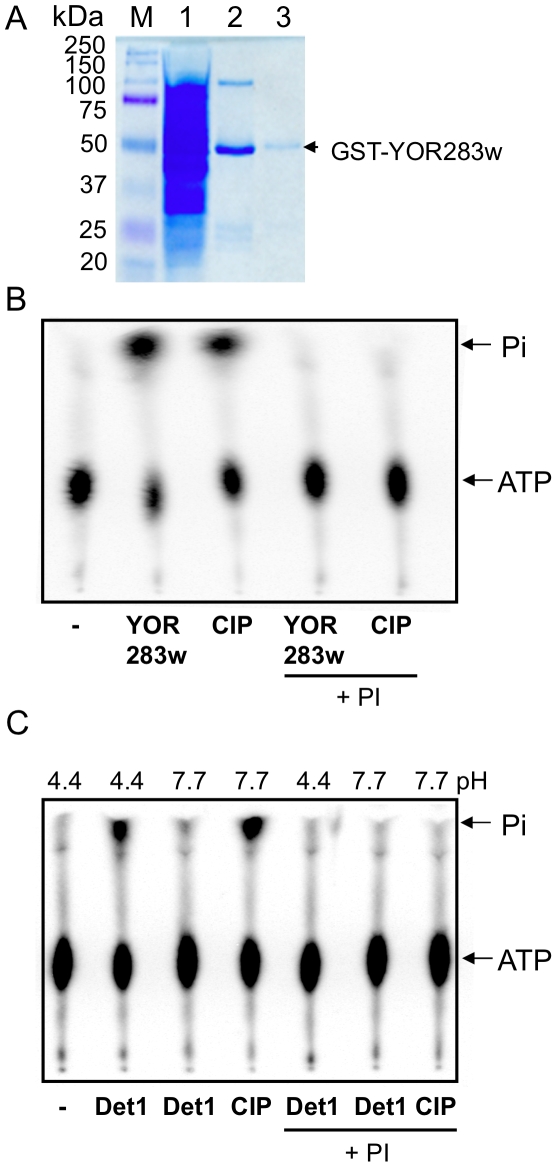
Phosphatase activities of Det1 and YOR283w. A. Purification of GST-YOR283w. Proteins were separated on a 12% SDS-polyacrylamide gel and stained with Coomassie blue. Lane M, molecular mass markers (kDa); lane 1, induced cell lysate; lanes 2 and 3, elution from glutathione agarose with 25 mM reduced glutathione buffer. Position of the GST-YOR283w fusion protein is indicated. Phosphatase activity of YOR283w using pH 7.7 buffer detected by TLC (B) or Det 1 at pH 4.4 or pH 7.7 (C). Reaction mixtures contain 250 ng GST-YOR283w, 5 µl aliquot of IgG sepharose-bound Det1 recombinant library protein or 1 unit of CIP (control), pH 4.4 or 7.7 buffers (50 mM) as indicated, supplemented with or without 1/100 phosphatase inhibitor (PI) cocktail set II (Calbiochem). Reaction mixtures were incubated at 37°C for 1 hr and reaction products were resolved by PEI-cellulose TLC plates. Positions of ATP and P_i_ are indicated.

Besides Pho8 and Ptc5, we detected phosphatase activities associated with ORFs YOR283w and YDR051c. As shown in Figure 4, active pools 45 and 132 containing YDR051c and YOR283w, respectively, both exhibited a signal corresponding to labeled phosphate. YOR283w encodes an uncharacterized protein while YDR051c encodes a protein called Det1, reported to have a cellular function in ergosterol transport between the endoplasmic membrane and plasma membrane [27]. Interestingly, a BLAST search analysis revealed that both proteins have an RHG motif in the N-terminal region, a conserved feature of the histidine phosphatase superfamily [28]. Many phosphoglycerate mutases (PGM) and phosphatases, such as the acid phosphatase Pho5 in budding yeast, belong to this superfamily [28]. To confirm the phosphatase activity of YOR283w, a GST-YOR283w fusion protein was expressed using a strain from a different library and was affinity-purified with glutathione sepharose beads to near homogeneity as described in Materials and Methods. The purified protein had an expected molecular mass of approximately 51 kDa (Fig. 5A). The GST-YOR283w fusion protein was incubated with [γ-^32^p] ATP and the reaction products were analyzed by TLC. The release of the labeled γ-phosphate from [γ-^32^p] ATP was blocked by the addition of phosphatase inhibitors, confirming that YOR283w possesses phosphatase activity (Fig. 5B).

Unfortunately, the protein expressed from the GST-Det1 strain from the GST-ORF protein fusion library was unstable and no activity was detected [29]. Therefore, the IgG Sepharose-bound Det1 was used in all experiments. Identity of the protein was confirmed by sequencing of the plasmid recovered from the MORF library strain, and the molecular mass was of the expected value of 58 kDa (with the tri-partite affinity tag) (data not shown). TLC was used to show that the sepharose bead-bound Det1 could remove γ-phosphate from [γ^32^p] ATP (Fig. 5C), confirming the phosphatase activity of Det1 detected using the oligonucleotide substrate (Fig. 4).

### Characterization of the Det1 and YOR283w Phosphatase Activities

**Figure 6:**
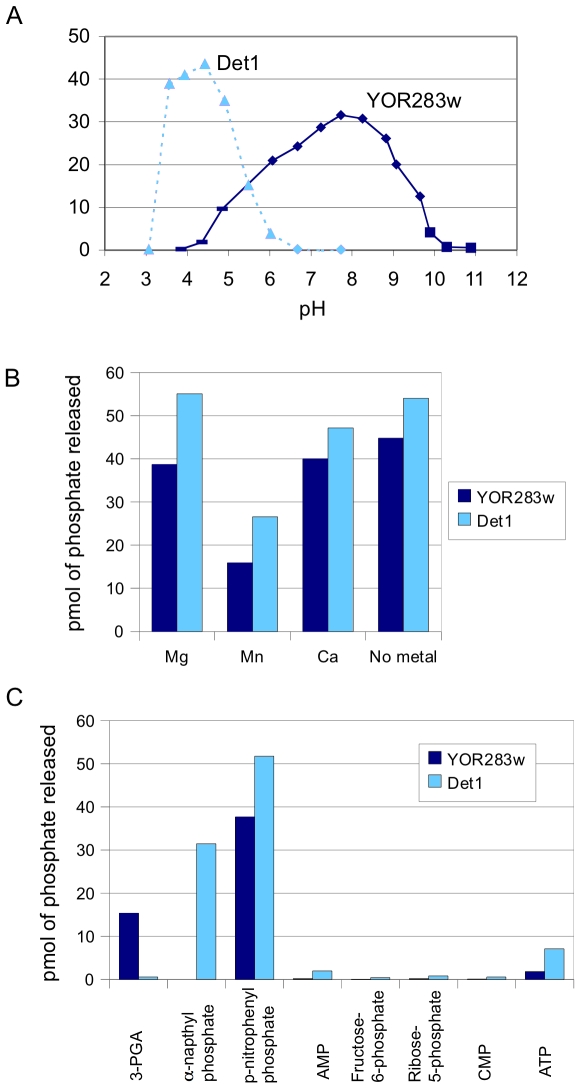
Characterization of the Det1 and YOR283w phosphatase activities. Optimum pH (A) metal dependence (B) and substrate specificity (C) of Det1 and YOR283w were measured by the release of inorganic phosphate as described in Experimental Procedures. σ, sodium acetate/acetic acid buffer; ⧫, Tris-acetate or Tris-HCl buffer; ν, CAPS/NaOH buffer.

The phosphatase activities of Det1 and YOR283w were further characterized with respect to optimum pH, substrate specificity and requirement of metal cofactors. To determine the pH optimums of both phosphatases, assays were carried out at different pHs, using ρ-nitrophenyl phosphate as the substrate. Det1 had a pH optimum of 4.5 (Fig. 6A), suggesting it is an acid phosphatase. TLC confirmed that Det1 removes inorganic phosphate from ATP only at pH 4.4 and not at pH 7.7 (Fig. 5C). In contrast, YOR283w exhibited a broad optimum pH between 7 and 9 (Fig. 6A). Our results also indicated that both phosphatases do not require divalent cations for activity as would be expected for members of the histidine phosphatase superfamily (Fig. 6B).

To explore the substrate specificity of Det1 and YOR283w, assays were performed with a range of phosphorylated substrates. Det1 was able to hydrolyze both hydrophilic and hydrophobic substrates such as α-napthyl phosphate, suggesting broad substrate specificity (Fig. 6C). On the other hand, YOR283w only showed phosphatase activity against hydrophilic substrates, such as 3-phosphoglyceric acid (3-PGA) (Fig. 6C).

## Discussion

The goal of this screen was to identify novel 5′–3′ exonucleases that degrade linear DNA and endonucleases that cleave branched DNA structures expected to formed during DNA repair or replication. At the time the screen was initiated, the identity of exonucleases involved in the 5′–3′ resection of DSB ends was unknown, and the only activities known to cleave DNA substrates containing a Holliday junction were the mitochondrial protein Cce1 and the Mus81-Mms4 complex.

From the screen of pooled strains we identified several known nucleases, including Rad27, Apn1, Pso2, Rex2 and Rat1. Although Rat1 and Rex2 are exoribonucleases, they appear to have activity on DNA substrates, similar to the Rat1-related protein Kem1/Sep1 [30], [31]. The 5′–3′ exonuclease Exo1 was not detected in the screen, but weak activity was found for the fusion protein individually purified (data not shown). The low activity could be because the protein is rapidly cleaved during extract preparation and the nuclease activity resides in the N-terminal region of the protein, not the C-terminal region that would have been recovered using the tag [32]. The failure to detect some known nucleases by the pooling strategy raises the possibility that unknown nucleases might have been missed in the screen if they have very low activity, or are inactive in the presence of 1 mM Mn^2+^ included in the reaction buffer. It is also possible that heteromeric nucleases would not be identified because only one component is over-expressed; however, we did identify a Rat1-associated protein, Rai1, in this screen. Of more concern is the lack of representation by nucleases in the library. The Dna2, Kem1 and Yen1 nucleases, as well as the catalytic subunits of DNA polymerase δ (Pol δ) and Pol ε, which have intrinsic 3′–5′ exonuclease activity, are absent from the MORF library. Dna2 has recently emerged as a candidate for 5′–3′ resection of the ends of DSBs by genetic and biochemical assays [6], [33], and Yen1 was identified as a HJ resolvase using the TAP-tagged strain collection [18]. Because the MORF library requires amplification of plasmids in *E. coli* it is possible that nucleases expressed at low levels in *E. coli* are toxic to the organism and thus are under-represented in the library. Most plasmid libraries are propagated in *E. coli recA* strains and low expression of a nuclease might be particularly toxic to *recA* mutants that have no capacity for homology-dependent repair.

Although no new nuclease activities were identified from this screen, we did find phosphatase activity associated with a protein of unknown function, YOR283w, and with the recently characterized protein Det1. Our preliminary biochemical characterization of these two proteins suggest they are able to hydrolyze phosphate from several substrates, including DNA, ATP, 3-PGA, α-napthyl phosphate and ρ-nitrophenyl phosphate, and that Det1 is active at acid pH whereas the phosphatase encoded by YOR282w has a broad pH range. Both proteins contain the conserved RHG motif characteristic of members of the histidine phosphatase superfamily, which includes phosphoglycerate mutases and phosphatases [28]. The conserved histidine residue is transiently phosphorylated during catalysis. Members of this family identified by sequence homology are frequently referred to as mutases, but most members are in fact phosphatases [28]. *DET1* was recently identified as a gene required for non-vesicular transport of sterols in both directions between the endoplasmic reticulum and plasma membrane [27]. The precise function in sterol transport is unknown, and our biochemical characterization does not identify potential substrates. A recent study identified the insect enzyme ecdysteroid phosphate phosphatase and the related human protein Sts-1 as members of the histidine phosphatase superfamily, and demonstrated phosphatase activity using ecdysteroid and steroid phosphate substrates [34]. Intriguingly, YOR283w was identified as essential for the viability of the *sec14 kes1* double mutant [35]. Sec14 is the major phosphatidylcholine (PC)/phosphatidylinositol (PI) transfer protein in budding yeast and is essential for vesicular transport from the Golgi apparatus. Kes1 is an oxysterol binding protein family member that binds to sterols and phosphatidylinositol 4-phosphate and regulates Golgi apparatus-derived vesicular transport. This finding raises the possibility that the physiological substrate for YOR283w could be a PI phosphate. Analysis of the activity of YOR283w and Det1 on specific phosphorylated lipids might be informative, as well as genetic studies to investigate the interaction with other components of the vesicular and non-vesicular transport pathways.

## Materials and Methods

### Purification of MORF library proteins

The biochemical screen used a *S. cerevisiae* MORF fusion protein library purchased from Open Biosystems [19]. Yeast strains from the library were first grown individually in synthetic complete (SCG)-URA liquid media in a 96-well plate format. After growth at 30°C for 2 days, pools of the library strains were grown in 200 ml synthetic medium containing 2% raffinose as a carbon source and lacking uracil (SCR-URA) until the culture reached an OD_600_ of 0.8 to 1.0. Fusion protein expression was induced by adding 100 ml 3xYP (3% yeast extract; 6% bacto-peptone) and 6% galactose, to a final concentration of 1xYP and 2% galactose, respectively. Expression was induced for 6 hours, cells were collected by centrifugation, washed with cold water, and stored at −80°C. To obtain crude lysates, cells were resuspended in 5 ml lysis buffer [20 mM Tris-HCl, pH 8.0; 300 mM NaCl; 1 mM DDT; 10% glycerol (v/v); 0.5 mM PMSF; 1/1000 protease inhibitor cocktail set IV (Calbiochem)]. An equal volume of acid-washed glass beads was added and cells were lysed by vortexing for 30 min at 4°C. The lysate was centrifuged at 14,000 rpm for 10 min. The supernatant was transferred to a clean tube and was centrifuged one more time. The cell extract was mixed with 200 µl IgG sepharose beads (GE Healthcare) and the mixture was gently rotated for 2 hrs at 4°C to adsorb proteins to the beads. The beads were collected by centrifugation, and the supernatant was discarded. The beads were washed 3 times with 50 vol of lysis buffer and 4 times with 50 vol of 3C protease cleavage buffer [50 mM Tris-HCl, pH 7.5; 150 mM NaCl; 10% glycerol (v/v)]. Beads with 4 vol of 3C protease cleavage buffer were incubated with ∼10 µg of purified GST-3C protease overnight. After centrifugation, the supernatant fraction, containing the cleaved proteins, and the sepharose bead-bound proteins were separately assayed for nuclease activities. The plasmid for expression of the GST-3C protease was a gift from E. Phizicky (U. Rochester) and was purified by glutathione agarose affinity chromatograpy from lysates of *E. coli*. To further characterize the phosphatase activity of Det1, IgG sepharose-bound Det1 was purified following this protocol using the individual library strain expressing Det1.

The *S. cerevisiae* GST-ORF fusion protein library was used to purify GST-YOR283w [29]. This library contains a collection of yeast strains, each expressing a yeast ORF fused at its N-terminus with a GST tag. The procedures of protein induction and cell lysis to obtain a cell extract were basically the same as the purification of the MORF library proteins. The extract was mixed with glutathione sepharose beads (GE Healthcare) for 2 hrs at 4°C to adsorb proteins to the beads. After the beads were precipitated by centrifugation and washed with lysis buffer, the GST fusion protein was eluted from the beads with 2 vol of 10 mM Tris-HCl, pH 7.8; 200 mM NaCl; 5 mM β-mercaptoethanol; 5% glycerol; 25 mM reduced glutathione. The eluted protein was dialyzed against 10 mM Tris-HCl, pH 7.6, 200 mM NaCl, 1 mM DDT, 50% glycerol, and stored at −80°C.

### DNA substrates

The synthetic Holliday junction (X26) substrate was constructed by annealing the following four oligonucleotides as described by Constantinou *et al*. (Constantinou *et al*., 2001).

Oligo 1:

5′-CCGCTACCAGTGATCACCAATGGATTGCTAGGACATCTTTGCCCACCTGCAGGTTCAC

CC-3′

Oligo 2:

5′-TGGGTGAACCTGCAGGTGGGCAAAGATGTCCTAGCAATCCATTGTCTATGACGTCAAG

CT-3′

Oligo 3:

5′-GAGCTTGACGTCATAGACAATGGATTGCTAGGACATCTTTGCCGTCTTGTCAATATCG

GC-3′

Oligo 4:

5′-TGCCGATATTGACAAGACGGCAAAGATGTCCTAGCAATCCATTGGTGATCACTGGTAGC

GG-3′

The 5′ end of Oligo 2 was labeled using [γ-^32^P] ATP and T4 polynucleotide kinase for all three substrates. The Y substrate was constructed by annealing Oligos 1 and 2 together. The Y and HJ substrate were gel purified following annealing. Oligo 2 was used directly as the ssDNA substrate in the screen.

### Nuclease assays

Each of the protein pools was assayed for nuclease activity using the 5′ end labeled 60-mer oligonucleotide, the Y-shaped substrate or the HJ-containing substrate. Reaction mixtures contained a 10 µl aliquot of the protein pool (cleaved proteins or beads), 20 mM Tris-HCl (pH 8.0), 5 mM MgCl_2_, 1 mM MnCl_2_, 1 mM ATP, 1 mM DDT and one of the three radiolabeled DNA substrates in a 50 µl reaction volume and were incubated at 37°C for 1.5 hr. The reactions were stopped with 10% EDTA, 0.2% SDS and 1 mg/ml proteinase K. Reaction products were resolved by 10% (for Y and HJ X26 substrates) or 15% (for ssDNA substrate) native polyacrylamide gels and analyzed using a phosphorimager (GE Healthcare). RecJ and T7 Exonucleases (New England Biolabs, Inc.) were used as controls for exonuclease assays, and RuvC was purified as described [36] and used as a control for HJ cleavage.

### Phosphatase assays

Phosphatase activity of Det1 and YOR283w was measured by monitoring the release of inorganic phosphate as previously described [37]. Reaction mixtures of 300-µl contained 250 ng GST-YOR283w (assay for optimum pH and metal dependence), 2.5 µl aliquot of IgG Sepharose-bound YOR283w fusion protein (assay for preferred substrates) or 5 µl aliquot of IgG Sepharose-bound Det1 library fusion protein (assay for optimum pH, metal dependence and preferred substrates) with different substrates (2.5 mM), divalent cations (5 mM), and/or buffer of different pHs (50 mM). Reactions were initiated by the addition of substrate (2.5 mM) and incubated at room temperature for 1 hr (assay for optimum pH) or at 37°C for 30 min (assay for metal dependence) or for 15 min (assay for preferred substrates). ρ-nitrophenyl phosphate was used as the substrate in the assays to determine optimal pH and metal dependence. Trichloroacetic acid (TCA) was added to the final concentration of 5% and reactions were centrifuged for 15 min for deproteinization. After addition of 700 µl molybdate reagent and incubation for 20 min at 45°C, the A_820_ was measured. All absorbance results were corrected for enzyme-unrelated substrate dephosphorylation.

For analysis of reaction products by thin layer chromatography (TLC), 300 µl reaction mixtures contained 250 ng GST-YOR283w, 5 µl aliquot of IgG sepharose-bound Det1 recombinant library protein or 1 unit of CIP (control), 1 pM of [γ-^32^p]ATP, different pH buffers (50 mM) and supplemented with or without 1/100 phosphatase inhibitor cocktail set II (Calbiochem) which contains imidazole, sodium fluoride, sodium molybdate, sodium orthovanadate and sodium tartrate. Members of the larger branch 1 of the histidine phosphatase superfamily are inhibited by vanadate, while members of the smaller branch 2 are inhibited by tartrate [38], [39]. Reaction mixtures were incubated at 37°C for 1 hr and reactions were stopped with 10% EDTA, 0.2% SDS and 1 mg/ml proteinase K. Reactions products were separated on PEI-cellulose TLC plates developed with 0.75 M KH_2_PO_4_, pH 3.5, and analyzed by a phosphorimager. Calf intestinal phosphatase (CIP) was used as a control for phosphatase assays.
